# Differences in sNPF Receptor-Expressing Neurons in Brains of Fire Ant (*Solenopsis invicta* Buren) Worker Subcastes: Indicators for Division of Labor and Nutritional Status?

**DOI:** 10.1371/journal.pone.0083966

**Published:** 2013-12-20

**Authors:** Paula Castillo, Patricia V. Pietrantonio

**Affiliations:** Department of Entomology, Texas A&M University, College Station, Texas, United States of America; Stanford University, United States of America

## Abstract

In the red imported fire ant, *Solenopsis invicta* Buren, the neuronal and molecular mechanisms related to worker division of labor are poorly understood. Workers from different subcastes (major, medium and minors) perform different tasks, which are loosely associated with their size. We hypothesized that the short neuropeptide F (sNPF) signaling system (NPY-like) could be involved in mechanisms of worker division of labor and sensing or responding to colony nutritional requirements. Thus, we investigated the expression of the short neuropeptide F receptor (sNPFR) in the brain and subesophageal ganglion (SEG) of workers from colonies with and without brood. Across worker subcastes a total of 9 clusters of immunoreactive sNPFR cells were localized in the brain and the subesophageal ganglion (SEG); some of these cells were similar to those observed previously in the queen. Worker brain sNPFR cell clusters were found in the protocerebrum near mushroom bodies, in the central complex and in the lateral horn. Other sNPFR immunoreactive cells were found at the edge of the antennal lobes. Across subcastes, we observed both a constant and a differential pattern of sNPFR clusters, with a higher number of sNPFR cells found in minor than in major workers. Those sNPFR cells detected in all worker subcastes appear to be involved in olfaction or SEG functions. The differential expression of clusters in subcastes suggests that sNPFR signaling is involved in regulating behaviors associated with specific subcastes and thus, division of labor. Some sNPFR cells appear to be involved in nutrient sensing and/or brood care, feeding behavior and locomotion. In colonies without brood, workers showed a lower cluster number, and an overall reduced sNPFR signal. Our results suggest the sNPF signaling system is a candidate for the neurobiological control of worker division of labor and sensing brood presence, perhaps correlating with protein requirements and availability.

## Introduction

The red imported fire ants (*Solenopsis invicta* Buren; Hymenoptera: Formicidae) are eusocial insects native from South America with an extraordinary capacity for adaptation to different environments. They have invaded countries such as the United States, Australia, New Zealand, China and Taiwan [1]. They are considered a dangerous invasive species in the U.S., affecting the habitat of other native animals [2]–[4]. Fire ant colonies are composed by individuals from different castes, designated as the reproductives (males and females) and the workers. This reproductive division of labor refers to specialization of drones and queens for the generation of new individuals, while female worker ants co-operate and perform brood rearing, care for the queen, forage for food, defend the nest, dig soil for nest construction, etc. The worker caste is composed of a greater number of individuals compared to the reproductive caste, and its members present large variations in body size. This size variation is known as worker polymorphism and in fire ants is the basis for the division of the worker caste into three loosely defined subcastes, as follows. Minor ants are the smallest workers, while major ants are the biggest. The third subcaste corresponds to medium workers of intermediate size between the former [5]. Previous studies showed that there is a correlation between the worker body size and the labor they perform, and it is believed this specialization of workers is necessary to increase their efficiency. However, other factors such as worker age influence task performed, making the prediction of worker task based on size less accurate. In the fire ants there are two main categories of workers, “nurses” and “foragers”, each group composed by a wide age/size range of ants. A third category of workers called “reserves” is very heterogeneous in age, size and behavior; they may work as nurses or foragers, or they may store liquid food, to which the name “reserves” refers to. In general, the smallest and youngest ants are responsible of taking care of the brood while older ants prefer to forage. However, foragers never feed larvae and nurses never forage [6]. In other ants and bees the endocrine mechanism appears to be related to a higher juvenile hormone titer in older workers that promotes foraging in comparison to younger nurses that remain in the nest [7]. Importantly, fire ant female workers are completely sterile, without ovaries, and thus are devoid of reproductive plasticity. Only queens can produce eggs. This makes this species a good model to separate female gene networks related to reproduction (queens) from those related to female worker tasks. Previous studies in other ant species have shown that the worker division of labor and the differences workers have when compared with the reproductive castes (drones, queens) are correlated with differences in the structure and organization of their brains [8]–[11]. For example, workers of several species of ants generally present small optic lobes and large antennal (olfactory) lobes compared with males and queens; and this correlates with the size of the mushroom bodies, especially in the latter input regions (lip and collar) [12], [13]. In carpenter ants, *Camponotus ocreatus*, mushroom bodies are significantly larger in workers than in queens and males; perhaps related to the fact that worker ants need to memorize the location of food sources and develop a good sense of spatial orientation to remember the way back to the nest. Both processes could be integrated in the mushroom bodies, which are the most important center of learning and information processing [14]. Other differences have been observed among ant castes, such as those in the patterns of serotonergic immunoreactivity in the optic lobe of *Pheidole dentata*, where old major workers exhibit an increased number of serotonergic cell bodies than old minors [15]. Also in those ants, and in agreement with these findings, serotonin titer increases in the brain with age, and major workers showed significant differences in the branching of serotonin-immunoreactive calyx input neurons than minors [16]. Considering all of the above, it is clear that ant behavior correlates with some physical differences in body size and changes of signaling molecules in the brain. Other factors such as ovarian activity [17] (but not in fire ants, as indicated above), genetics [18], [19], and even patterns of DNA methylation [20] could modify the division of labor among worker ants.

Neuropeptides could regulate behavior through the temporal and spatial coordination of several neuronal circuits that could involve the participation of sensory neurons, interneurons and motor neurons [21]. In most arthropods studied so far, short neuropeptides F (sNPFs) are 6–11 amino acid residues in length with the C-terminal consensus sequence xPxLRLRFamide. These neuropeptides are important because are involved in the regulation of several critical functions such as: feeding and growth, stress responses, locomotion, olfaction, hormone release, reproduction, learning and memory [22]. The short neuropeptide F (sNPF) exerts its action through the short neuropeptide F receptor (sNPFR), a G protein-coupled receptor (GPCR) related to the mammalian NPY receptor (Y2). The sNPFR was first identified and characterized in *Drosophila*, where just one variant of the receptor (sNPFR1) was found in brain and diverse tissues [23].

In animals, NPY signaling may play a role in the motivation towards foraging behavior [24]. In honey bees the NPY system is apparently represented only by the sNPF (sNPY) signaling system because the long NPF receptor is absent from the genome although the long NPF (NPY) peptide is present; it is yet unknown if both peptides activate the same receptor [25]. In honey bees the sNPF signaling system is involved in the regulation of foraging behavior and its receptor transcript expression is higher in foragers than nurses, and lower in workers well fed with sugar and pollen (where pollen is a protein source), than in food deprived workers (two days with honey followed by two days starvation) [25]. Foragers exhibit higher levels of sNPFR transcripts when given a poor diet of sugar in comparison with younger bees and nurses feeding the same diet [25]. The higher sensitivity of foragers to nutritional cues could be mediated through sNPF and sNPFR signaling [25]. In agreement, in bees the sNPF peptide level varied in workers collecting either nectar or pollen and between foragers arriving or departing from a feeder, and these latter changes in peptide expression were highly dynamic, within minutes, supporting the sensitivity of the sNPF peptide expression to nutrition and foraging [26]. In fire ant queens we previously cloned the sNPFR cDNA; the receptor transcripts were highly expressed in brain and other tissues [27]. We were the first to show that the expression of the sNPFR transcript was significantly reduced in the brain of queens in response to starvation when they were provided only water, suggesting this receptor could be involved in the regulation of feeding behavior because its transcript level was affected by variation in the nutritional status [27].We later published a detailed description of the localization of 164 sNPFR-immunoreactive cells in the queen brain [28].

Here we focused on the immunolocalization of the short neuropeptide F receptor (sNPFR) in the brain of fire ant workers, investigating if there is a relationship between the sNPF/sNPFR signaling pathway and worker division of labor (subcastes), and their sensing/or responding to colony nutritional requirements, which could be affected by the presence or absence of brood. Protein digested by larvae provides amino acids that are used for further brood growth which results in colony growth during the summer, while carbohydrates are the preferred energy source for the colony [29]. In summary, in fire ants presence of larvae reflects a demand for amino acids [30]. Based on the dynamic changes found in the sNPF peptide in honey bee worker brains in response to nutritional cues (pollen vs. nectar) and foraging behavior, herein we investigated the distribution of the sNPFR in the brain of fire ants workers in colonies with and without brood. Our hypothesis states that changes in protein requirements and/or availability as consequence of the presence of hungry larvae would be reflected centrally in the sNPF signaling system in workers, specifically in sNPFR expression. These experiments were planned to reveal functions of sNPF signaling in sterile workers, and thus unrelated to reproduction at the individual level, but relevant to worker tasks and colony growth status and nutritional requirements.

## Materials and Methods

### Insects

Fire ants are invasive in Texas and ubiquitous. Polygyne colonies of *S. invicta* were a gift of Dr. R. Puckett at Texas A&M University. All colonies were collected at the “5-Eagle Ranch” (30°37′49.92″N; 96°40′19.37″W) in Burleson County, Texas, from May to July 2012. The Ranch owner authorized Dr. Puckett to collect fire ant colonies which are unwanted in the property. The field collections were limited to fire ants and did not involve endangered or protective species. Colonies with and without brood (egg and larvae) were used, and all of them had mated queens. Colonies without brood were those which queens have had the capacity to lay eggs, as observed for months previously, but had stopped producing brood. All the colonies were maintained in the laboratory on plastic trays, whose walls were covered with Fluon (Insect-a-slip©, BioQuip products, CA, USA) at 27±2°C in a 12∶12 h light- dark photoperiod. Each plastic tray contained at least one nest (10 cm diameter Petri dishes half-filled with Castone® (Dental Supply International Inc., York, PA, USA). The ants were fed daily with 15% honey-water and frozen crickets (Fluker's Cricket Farm, Port Allen, LA, USA). Water was provided *ad libitum*.

### Classification and selection of worker ant subcastes

**Figure 1:**
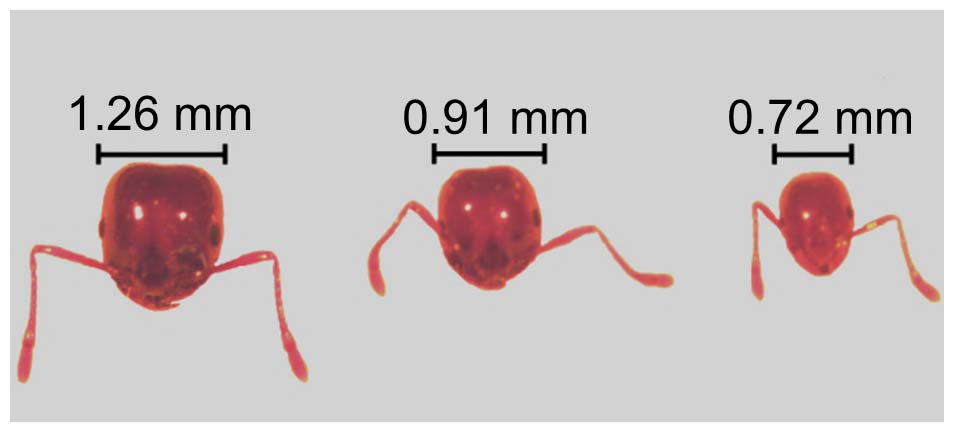
Comparison of the head widths (H.W.) among worker subcastes in fire ants. Majors are considered the biggest workers, minors are considered the smallest, and mediums are intermediate in size between majors and minors. The heads of a major worker (left; 0.93–1.46 mm H.W.), a medium worker (center; 0.73–0.92 mm H.W.) and a minor worker (right; ≤ 0.72 mm H.W.) are shown.

Worker ants were collected from colonies with and without brood, and classified into majors, mediums and minors according to the head width (H.W.) as described previously by Wilson [5]. In Wilson's study, the distribution of sizes of the head width (H. W.) was determined in the range from 0.48 to 1.46 mm. Minor workers are considered the smallest members of the worker caste, with H. W≤0.72 mm; medium workers have a H.W  =  0.73–0.92 mm, and in major workers the H.W is ≥ 0.93 mm [5]. The differences in H.W. we observed in our colonies are shown in Figure 1. Major workers were collected on the tray open areas, far from the queen nest, or collected on the small food dishes were the crickets were provided. Medium workers were collected on the way in/out the nest, or on the surrounding open areas outside the nest. Minor workers were collected from inside the nest, around the mated queen or carrying brood. Depending on the different tasks workers were performing when collected for dissection, we considered the major workers as “foragers” and minor workers as “nurses”. The medium workers selected, however, were not performing any specific task, sometimes they were found inside the nest carrying brood, outside the nest foraging, or just standing outside the nest.

### Dissection of the worker ant brains and subesophageal ganglion (SEG)

All worker ants were dissected using the same procedure, as described below. Selected ants were anesthetized on ice for about 10 min; the head was cut off and placed on a dissection dish with silicone on the bottom (Sylgard®, Dow Corning Corporation, Midland, MI, USA). Then, using thin forceps, antennae were removed by pulling and the head was held through the mouth. Several small punctures were made on the sides of the head above the mandibles, using a fine dissection pin (#2). After breaking the cuticle, PBS was added to the dissection dish, until the head was completely submerged. Using thin forceps, the cuticle was removed very carefully, starting from the previously made punctures. With a spooning movement of the closed forceps all tissues inside the head capsule were removed carefully including esophagus, tracheae and glands, with the brain/SEG being enclosed in these tissues and rarely easily observed. The brain and SEG were finally exposed by removing the surrounding tissues under buffer. All the brains/SEG were collected individually in Eppendorf tubes for fixation that was performed using 4% paraformaldehyde/PBS solution at 4°C for 2 h.

### Antibodies

To immunolocalize the cells expressing the sNPF receptor in the fire ant worker brain, we used the same specific anti-peptide antibody developed against the *S. invicta* sNPFR described previously by Lu *et al.* and used for receptor expression analyses in brain and ovaries of fire ant queens [28].

The hydrophilicity and antigenicity profiles of sNPFR amino acid sequence (GenBank: DQ026281) had been analyzed using DNASTAR and ExPAsy software to determine and select antigenic regions for anti-peptide antibody production, and the sequence “CRGDKIDNGNNTMQETL” was selected for antibody production. This amino acid sequence is located toward the receptor C-terminus encompassing residues 331 to 347. The polyclonal and affinity purified antibody was developed by Pacific Immunology (CA, USA) in New Zealand female rabbits, using the synthetic peptide conjugated with keyhole limpet hemocyanin (KLH). After purification, the specificity of the antibodies was verified by ELISA (tested by Pacific Immunology, CA), and additional characterization of this antibody was performed by western blot as described by Lu *et al* [28].

### Immunolocalization of the sNPFR on brains/SEG of worker ants

From colonies with brood, 40 brains from majors, 52 each from mediums and minors were dissected, processed and analyzed for sNPFR immunoreactivity. Brains for negative control treatments were additionally dissected. From colonies with brood, a minimum of 6–10 brains were used per subcaste for either preimmune or antigen-preabsorbed antibodies (12–20 total per subcaste). Initially, additional negative controls were run with secondary antibodies only (about 4 brains per subcaste, not shown). From colonies without brood, 26 brains from majors, 26 from mediums and 16 minors were processed and analyzed for sNPFR immunoreactivity. For negative controls for all subcastes four brains each for preimmune, preabsorbed and secondary antibody only were dissected and processed (n = 12 per subcaste). In summary, a total of at least 284 brains were used in this study. In all cases half of the brains for all treatments from colonies with or without brood were mounted for analysis of either anterior or posterior brain view. Due to the time consuming task of selecting workers performing specific behaviors followed by brain dissection, the results were obtained through ten independent experiments, each with negative controls.

The expression of the sNPFR in brains/SEG of workers in all subcastes (minors, mediums and majors) was determined by whole mount immunofluorescence as described previously [28]. Briefly, after fixation, the brains/SEG was washed with 70% ethanol on ice. Then, the tissues were rinsed with PBST (PBS with 0.1% Tween) and incubated with 12 µg/ml protease K (Sigma-Aldrich, St. Louis, MO, USA) in PBS for 10 min. Both steps were performed at room temperature. The protease was removed by washing with PBST and then, the tissues were blocked for 24 h at 4°C using PBST with 10% normal goat serum (NGS) (Jackson ImmunoResearch, West Grove, PA, USA). After blocking, tissues were incubated with a 2 µg/ml primary antibody solution containing 2% NGS in PBST for 48 h at 4°C. A goat anti-rabbit IgG conjugated with Alexa Fluor® 546 dye (Invitrogen™) was used as secondary antibody (10 ng/ml) in the same solution as above Finally, the brains/SEG were mounted on glass slides using Vectashield™-DAPI (Vector, Burlingame, CA, USA) for nuclear staining. Cover slips for tissues examined under microscopy were 0.16–0.19 mm thick (No. 1.5; Fisher Scientific).

To ensure the specificity of the primary antibody, negative control tissues were included in this study as follows: antigen pre-absorbed anti-sNPFR antibody (500 µg of peptide antigen was incubated with 4 µg in one ml solution and diluted 1∶2 for final use); pre-immune rabbit serum (1∶1000 dilution) instead of primary antibody; and tissues incubated with secondary antibody only (10 ng/ml; 1∶200 dilution of commercial product).

### Data collection and analysis

Worker ant brains were analyzed for immunofluorescence from the anterior and posterior views. The anterior view corresponds to the frontal side of the brain, where the antennal lobes are clearly seen and oriented forward; while the posterior view corresponds to the back side of the brain/SEG were the subesophageal ganglion is more prominent. The tissues were analyzed using a Carl Zeiss Axioimager A1 fluorescent microscope, coupled with an AxioCam MRc color camera (Carl Zeiss). These images were captured and processed using the Axiovision software (version 4.8.2) provided with the microscope. All pictures were taken at a resolution of 1388 x 1040 pixels and were saved as TIFF files. No colored images were used, and in some images brightness and contrast were adjusted to clearly show the fluorescent signal found on the tissues by using the shading correction tool provided in the Axiovision software. For this, the whole image was corrected to improve image quality with no partial sections of images modified. Schematics were manually drawn and colored using PowerPoint software (Microsoft™).

## Results

### Characteristics of the immunostaining pattern of sNPF receptor (sNPFR) in brain and subesophageal ganglion (SEG)

The number of individuals analyzed is comparable to previous studies of insect NPY-signaling system [24]. Majors had an average brain width (B.W.) of 668.46 µm, medium workers 602.39 µm and minors have a B.W. of 573.30 µm in average. A total maximum of 9 clusters of cells expressing the sNPFR are present across worker subcastes in the fire ant. Figures shown are representative of the staining that was very consistent across subcastes within colonies with or without brood. When any small variation was observed, this is specifically mentioned.

**Figure 2:**
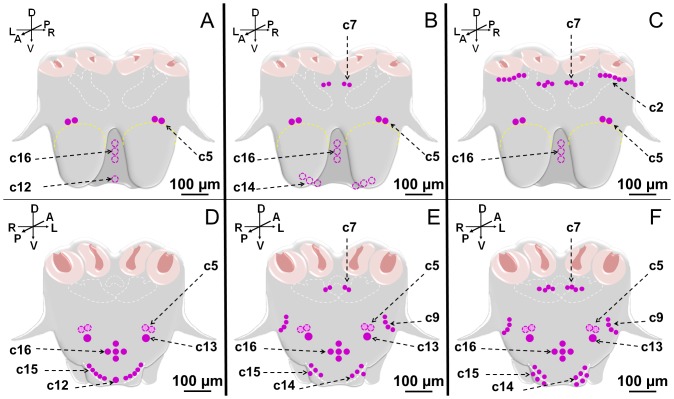
Schematic representation of the sNPFR immunolocalization in the brain and SEG of all worker subcastes. Anterior (top panel) and posterior (bottom panel) views of the brain show different cell clusters expressing the sNPFR. (A, D) represent the localization in majors; (B, E) in mediums and (C, F) in minors. Dashed-empty circles indicate the cells can be observed faintly from the anterior view but are located deeper in the brain; purple checkered-filled circles indicate the same, but when the brain is seen from the posterior side. Within clusters the depth of certain cells may vary. The brain orientation is indicated by the direction of the arrows shown in the top left corner of each subpanel: A =  anterior, P =  posterior, D =  dorsal, V =  ventral L =  left, R =  right.

We observed consistent differences among subcastes as defined (Figure 1), in the number of immunolabeled clusters present, with certain clusters being present only in one subcaste. A schematic representation summarizing the location of the cell clusters expressing the sNPFR in majors, medium and minor workers, from the anterior and posterior views of the brain is showed in Figure 2. Different cell clusters could be observed only from either the anterior or posterior view of the brain and others from both, providing subjective but relevant information about their depth relative to the surface of the brain observed. The anterior view of the brain shows the antennal lobes toward the front; and the posterior view shows the SEG towards the front (Figure 2). In the queen brain twelve clusters designated C1- C12 were previously reported [28]. Some of the cell clusters expressing this receptor in fire ant worker brains were highly reminiscent of those observed in the queen brain. This similarity determined that the number assigned to these clusters was retained for the worker brain but utilizing the small letter c (for cluster) followed by the same cluster number observed in the queen. We identified in workers as a group five of these apparently common clusters with the queen: c2, c5, c7, c9 and c12. However, except c5 which is present in all subcastes, the rest are differentially present among worker subcastes. Novel clusters found exclusively in workers were numbered c13-c16 (Figure 2; Table 1).

**Table 1:** Number of sNPFR immunoreactive cells found in the brain of worker subcastes from colonies with and without brood and comparison to those reported previously for queens.

	Workers from colonies with brood						Workers from colonies without brood						Queens from colonies (2)	
	Majors		Mediums		Minors		Majors		Mediums		Minors			
Cluster	Cell N per brain/SEG hemisphere (1)	Total N of cells per brain	Cell N per brain/SEG hemisphere (1)	Total N of cells per brain	Cell N per brain/SEG hemisphere (1)	Total N of cells per brain	Cell N per brain/SEG hemisphere (1)	Total N of cells per brain	Cell N per brain/SEG hemisphere (1)	Total N of cells per brain	Cell N per brain/SEG hemisphere (1)	Total N of cells per brain	Cell N per brain/SEG hemisphere (1)	Total N of cells per brain
C1 ^a^	–	–	–	–	–	–	–	–	–	–	–	–	N/A	3
^*^C2 ^b^	–	–	–	–	4–10	8–20	–	–	–	–	–	–	25	50
C3 ^a^	–	–	–	–	–	–	–	–	–	–	–	–	8	16
C4 ^a^	–	–	–	–	–	–	–	–	–	–	–	–	6	12
C5 ^c^	2–3	4–6	2	4	2	4	2	4	2	4	2	4	3	6
C6 ^a^	–	–	–	–	–	–	–	–	–	–	–	–	N/A	30
^*^C7 ^d^	–	–	2–4	4–8	4	8	–	–	–	–	–	–	4	8
C8 ^a^	–	–	–	–	–	–	–	–	–	–	–	–	11	22
^*^C9 ^d^	–	–	4	8	4	8	–	–	–	–	–	–	4	8
C10 ^a^	–	–	–	–	–	–	–	–	–	–	–	–	1	2
C11 ^a^	–	–	–	–	–	–	–	–	–	–	–	–	1	2
^*^C12 ^e^	N/A	2–3	–	–	–	–	–	–	–	–	–	–	N/A	5
c13 ^f^	1	2	1	2	1	2	1	2	1	2	1	2	–	–
c14 ^f^	–	–	2–3	4–6	3	6	–	–	2–3	4–6	3	6	–	–
^*^c15 ^g^	3–5	6–10	1–3	2–6	3	6	1–2	2–4	–	–	–	–	–	–
c16 ^f^	N/A	5	N/A	5	N/A	5	N/A	5	N/A	5	N/A	5	–	–
**Total cell number (range)**		19–26		29–39		47–59		13–15		15–17		17		164
**Percent change in cell N**° (3)								32–42		48–56		63–71		

Footnotes: Names of clusters in capital letters correspond to those in the queen, but cell numbers under minor, mediums and majors correspond to worker clusters in similar position to those clusters found in queens and that we identified with small letter c throughout the manuscript. Note that clusters only present in the midline of the brain, and therefore not symmetrically distributed are: C1, C6, C12 and c16; clusters c13 through c16 are exclusively found in workers, but c14 is absent in majors.

(1) The number of cells per cluster in one brain/SEG hemisphere is indicated only for clusters that show a symmetrical distribution; in workers, numbers separated by a hyphen indicate the range in the number of cells observed in different individuals. N/A refers to clusters in the midline of the brain which cell number is only indicated in the total number per brain column.

(2) From Lu *et al* [28].

(3) The percentage decrease in the cell number range per subcastes in colonies without brood was calculated with respect to the respective range in cell numbers in colonies with brood.

(a) are queen exclusive clusters;

(b) minors and queen exclusive;

(c) is a cluster common to all females (all worker subcastes and queen);

(d) common to mediums, minors and queens;

(e) queen and majors exclusive. In workers, clusters labeled c or f are immunostained regardless of the presence or absence of brood (C5 and c13, c14, c16). In workers, clusters with asterisks change in cell number depending on the presence or absence of brood; c15 is the only worker exclusive cluster that responds to the absence of brood (g).

An important observation is that the total number of cells expressing this receptor decreases from minor to major workers (Figure 2). The higher number of cells in minors is due both to a larger number of cell clusters and to the presence of more cells in the specific cluster. Major worker ants exhibited a total of five cell clusters (19–26 sNPFR immunolabeled cells), while minor workers showed eight clusters (47–59 sNPFR immunolabeled cells). Medium workers were intermediate in immunolabeled cell number, having seven clusters of cells (29–39 sNPFR immunolabeled cells) (Figure 2; Table 1). While the location and the characteristic cell number of certain clusters (c5, c13, c16) was constant in all worker subcastes (perhaps except for c5 in majors with some individuals showing increased number from 4–6 cells vs. 4 in others) in colonies with or without brood (Table 1), others showed an apparent increasing gradient in the number of cells from majors to minors such as cluster c7, absent in majors, with 2–4 cells in each side of the brain in mediums and four in each side in minors (see Figure 2, D–F, Table 1).

### Distribution of the sNPFR in the brain superior protocerebrum in worker subcastes

**Figure 3:**
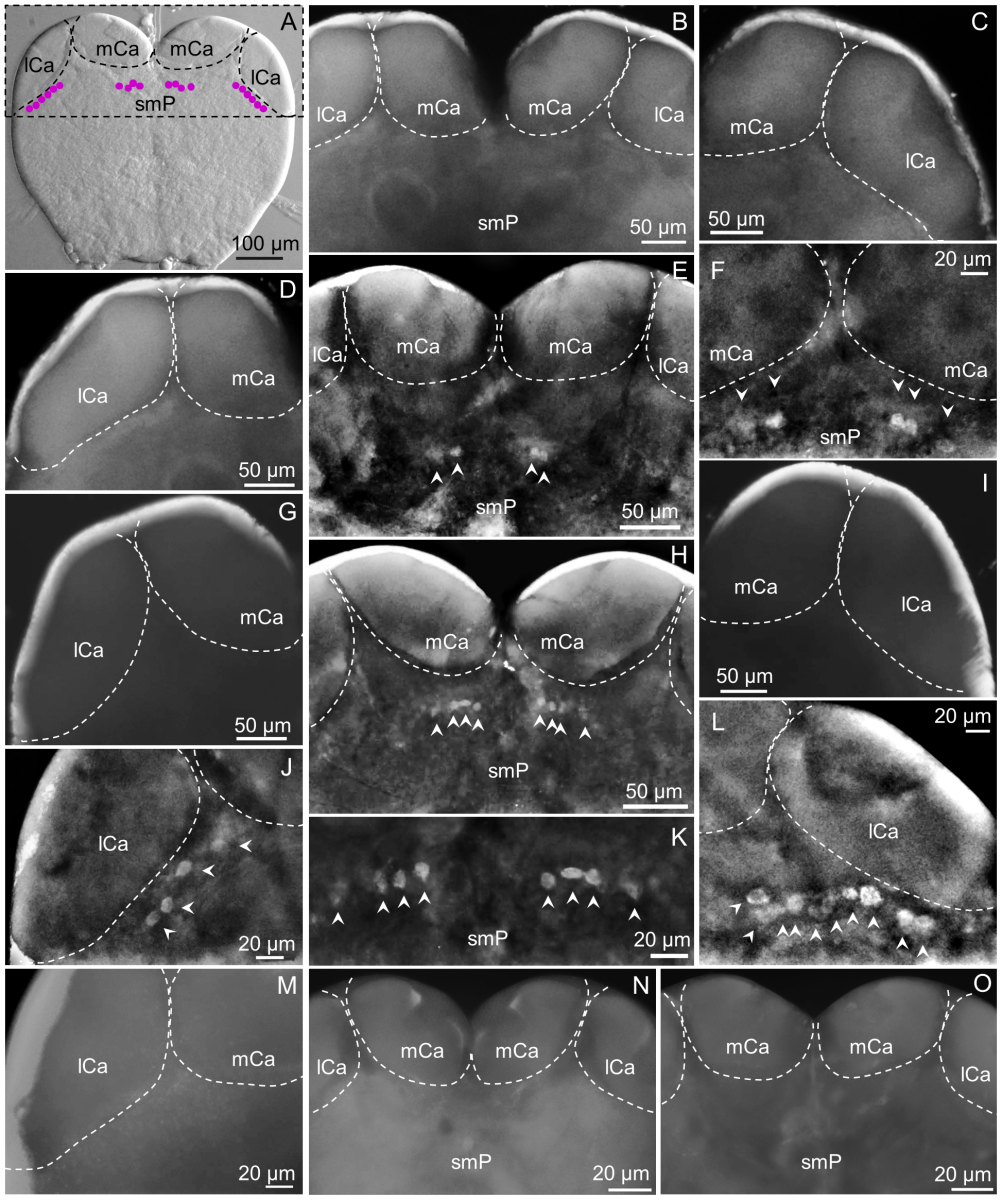
The superior protocerebrum exhibits differential sNPFR immunoreactive neurons among subcastes (anterior brain view). A: Nomarsky image of a minor worker brain showing the location of clusters c7 (center) and c2 (lateral). In majors, clusters c7 (B, center), and c2 (C, right; D, left) were not detected. E: shows c7 in the brain of a medium worker (detail in F); however, cluster c2 normally under the lCa is not observed (G, left; I, right). In minors, c7 is clearly visible close to the mCa (H, detail in K) and c2 is located under the right (L) and left lCa (J). The lack of fluorescent signal on the left lCa of a minor worker brain treated with pre-absorbed antibody as negative control is shown in (M), and both brain hemispheres are shown in (N). No fluorescent signal was observed for the pre-immune negative control (O). mCa: median calyces, lCa: lateral calyces, smP: superior medial protocerebrum. Left or right refers to the brain hemisphere shown.

The most remarkable differences among subcastes in the pattern of cells expressing the sNPFR were observed in this region of the brain (Figure 3). In Figure 3A a summary of immunoreactive cells that could be collectively found in minors and mediums, c2 (lateral) and c7 (center), are shown. In major workers these were not found (Figure 3, B–D). Cluster c7 is located above the fan-shaped body (FB) of the central complex in the superior medial protocerebrum (smP), under the medial calyces (mCa) of the mushroom bodies (Figure 3, E, F, H, K).

In medium workers c7 was observed from both brain views (anterior, Figure 2, B, and Figure 3, E, F, and posterior, not show, but see Figure 2, E) and the number of cells in this cluster was variable, from two to four cells. Also, not always both groups were identical in the same individual, as shown in Figure 3, F, where three cells are clearly labeled on the right side and two on the left side of the brain. In mediums, similarly to majors, c2 is completely absent (Figure 3, G, I).

In minor workers, clusters c2 and c7 were present, and c2 could be observed only from the anterior view of the brain. Cluster c2 is located right below the lateral calyces (lCa) of the mushroom bodies, symmetrically on both brain hemispheres and the number of cells in each lateral cluster varies from 4 (Figure 3, J) to 10 cells (Figure 3, L). Also, the shape and size of the cells in this cluster is variable, and some of them seem to be located deeper into the brain. Cluster c7 could be observed from both views, in the same location observed in medium workers (Figure 3, H) but this cluster was always observed as two groups of four cells each, similar in size and shape (Figure 3, H, K). No labeled cells were observed in negative controls of all subcastes, as expected (not all shown) (Figure 3, M–O).

### Distribution of the sNPFR in the brain central region in worker subcastes

**Figure 4:**
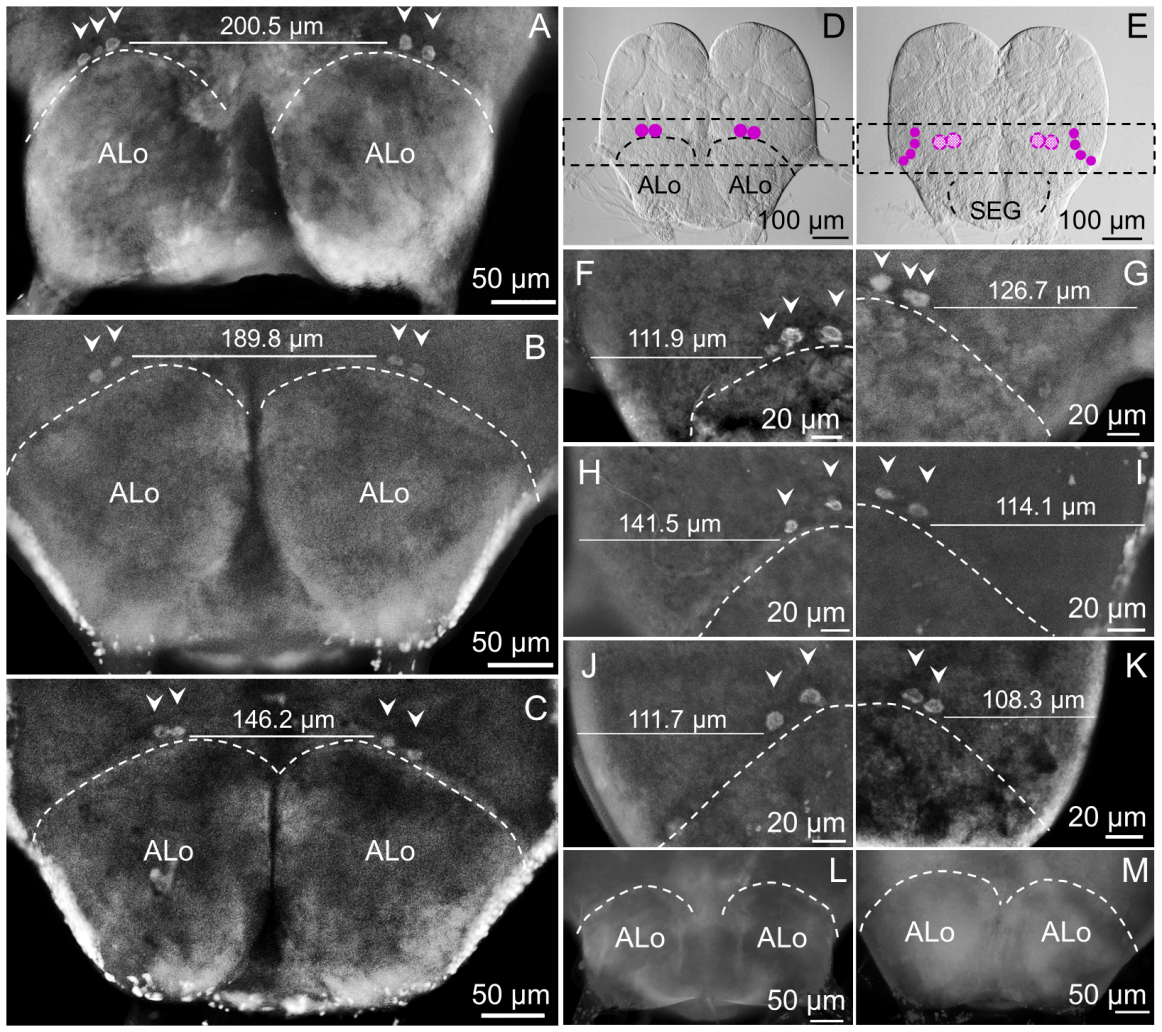
Cluster c5 is immunolocalized at the superior edge of the antennal lobe in all subcastes. Cluster c5 could be observed in the brain of majors (A), medium (B) and minor (C) workers more clearly from the anterior view but also from the posterior view, slightly deeper in the brain. A schematic representation is shown over a Nomarsky image of a medium worker brain from the anterior view in D. Cluster c5 is most often represented by two cells, which are symmetrically located at the superior edge of the ALo. In the posterior view of the brain in E, c5 is depicted using checkered dots; purple solid dots correspond to cluster c9. Cluster c5 in majors is shown in detail in F (left), G (right); notice that in those images c5 is composed of three cells. H (left), I (right) show a detail of c5 in mediums, and the same is shown in J (left), K (right) for minors. No fluorescent signal was observed in negative controls with pre-immune serum (L) or with the antigen-preabsorbed antibody (M). Left or right refers to the brain hemisphere shown.

**Figure 5:**
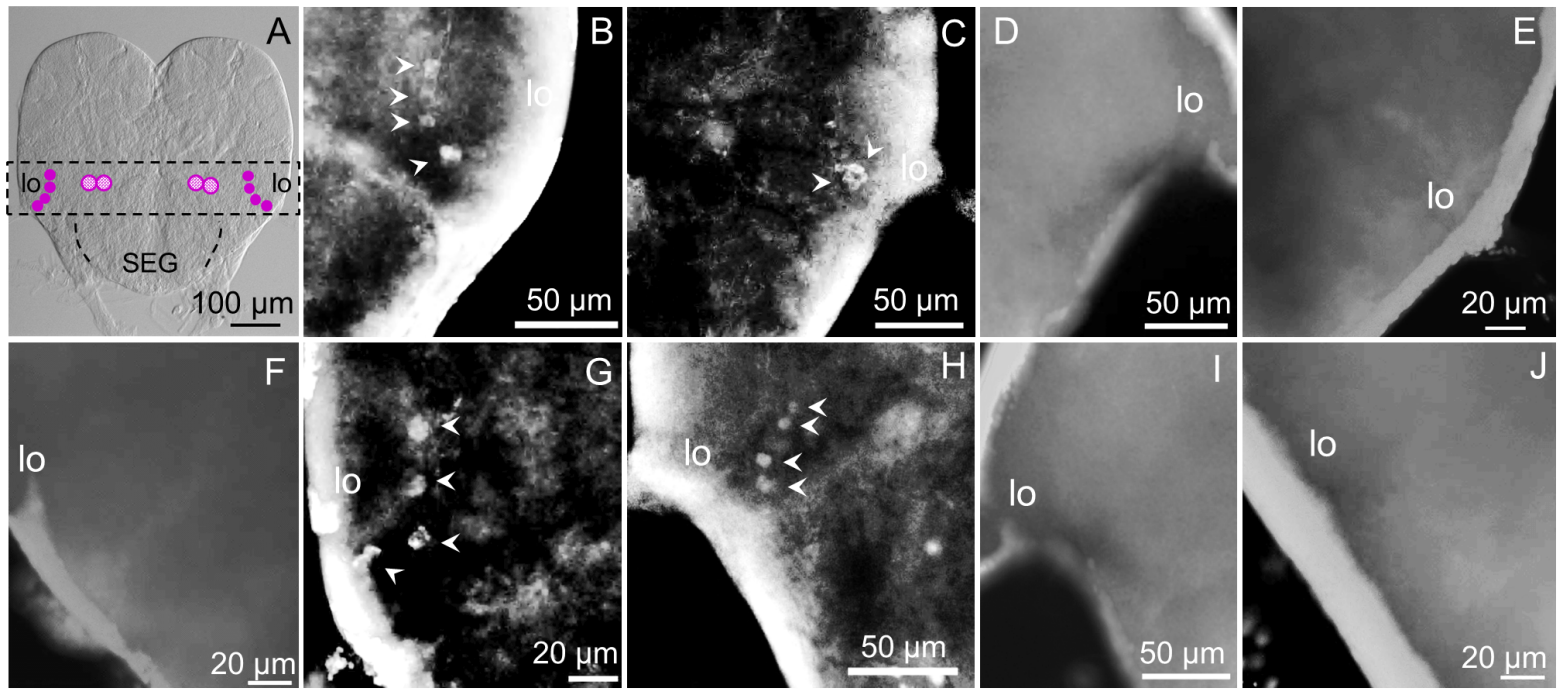
sNPFR immunoreactive cluster c9 is only detected in the posterior lateral protocerebrum of mediums and minors (posterior view). (A) Schematic representation of the location of c5 (central) and c9 (lateral). Images in the top panel show the right hemisphere and the bottom images, the left one. In minor worker brains cluster c9 is detected near the optic lobe, symmetrically on both sides of the brain (B, G). Also, c9 is present in medium worker brains (C, H). In majors, cluster c9 is undetectable (D, I). No fluorescent signal was observed in negative controls, with pre-immune serum (E, F) or antigen-preabsorbed antibodies (J). Lo: lobula.

This region includes the inferior protocerebrum and the area corresponding to the superior edge of the antennal lobe above the deutocerebrum (if observed from the anterior view where cluster c5 is detected (Figure 4)), or corresponds to the superior commissure of the SEG (if observed from the posterior view where cluster c9 is detected (Figure 5)).

From the anterior view, cluster c5 can be seen in the brain of major (Figure 4, A), medium (Figure 4, B) and minor workers (Figure 4, C). Cluster c5 is composed of two strongly labeled cells, located symmetrically and horizontally aligned on the superior edge of each antennal lobe as shown in the schematic (Figure 4, D. Purple dots); in some majors the two cells appear to touch each other while in other individuals they are ∼10 microns apart (Fig. 4A). Viewed from the posterior side, these cells appear to be located deeper in the brain than when viewed from the anterior side (Figure 4, E. checkered-filled dots). Sometimes a third cell could be observed in c5 in majors (Figure 4, F, G), but only two cells were observed in mediums (Figure 4, H, I) and minors (Figure 4, J, K). Representative negative controls (Figure 4, L, M) did not show any immunoreactivity, as expected.

From the posterior view of the brain, cluster c9 is observed in the inferior lateral protocerebrum, near the lobula of the optic lobe (Figure 5, A). This cluster is present in minor (Figure 5, B, G) and medium workers (Figure 5, C, H), but is not detected in majors (Figure 5, D, I). Usually this cluster is observed as four cells similar in size and shape, with cells distributed vertically forming a curved line (Figure 5; B, G, H). Nevertheless, sometimes immunoreactive cells in this brain region appear to be closely grouped in a circular fashion, just as in the brain of mediums shown in Figure 5 C.

### Distribution of the sNPFR on the posterior-inferior brain and SEG in worker subcastes

**Figure 6:**
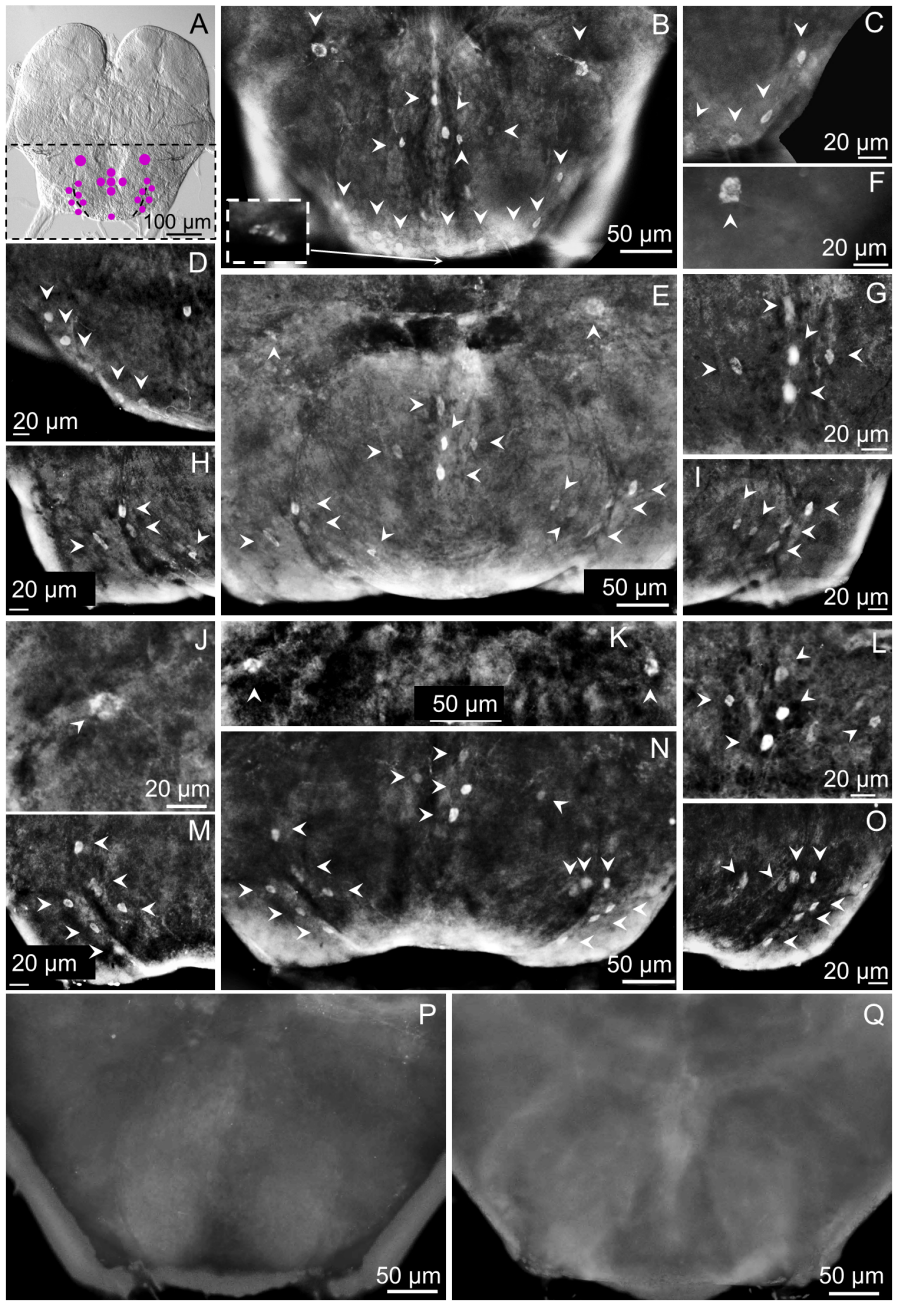
Common and subcaste-differential sNPFR immunoreactive clusters are present in the posterior brain and SEG. A schematic of all possible clusters detected in the posterior SEG across subcastes are shown in (A) over a Nomarsky image of a major. In all subcastes clusters c13 (top arrows in B), c15 (B, bottom arrowheads) and c16 (B, center) are present. Clusters c12 and c14 are differentially detected among subcastes. (B) In majors, c12 (dashed inset, arrow) is present but c14, that should be located internal to c15, is not detected. A detail of c15 in the right hemisphere of majors is shown in (C) and left, in (D). Medium and minors exhibit all clusters except c12, but although c14 and c15 are present, they have reduced cell number with respect to those in majors (compare D with H and M). In these two subcastes c14 and c15 could be observed from both sides of the brain. (E) Medium worker, clusters c13 to c16; details in F (c13), G (c16), H (c14, c15, left) and I (c14, c15, right). In minors, c13 is shown in (J), and in both brain hemispheres, in (K). (L) Brain of minor; sometimes cells in c16 are not perfectly distributed in a cross-like pattern (compare with G). (N) Distribution of clusters c13 to c16 in minors. Clusters c14 and c15 are show in M (left) and O (right). No fluorescent signal was observed in negative controls with pre-immune serum (P) or antigen-preabsorbed antibodies (Q).

This region of the brain contains the largest number of cells expressing the sNPFR in worker ants of all subcastes (Figure 6, A). Across all subcastes, clusters 12–16 are present (Figure 2, D–F); however, there are differences among subcastes. In majors, clusters c12, c13, c15 and c16 are observed, while c14 is not detected (Figure 2, D; Figure 6, B–D). In mediums and minors c13-c16 are present while c12 is not detected (Figure 2, E, F and Figure 6, E–O).

Cluster c12 is formed by a group of two or three cells located at the center and bottom edge of the SEG (Figure 2, D and Figure 6, B, see arrow and dashed inset). Cluster c13 is present in workers of all sizes, and is composed of two very large cells (average 13.9 µm diameter each) located symmetrically, apparently at the upper commissure of the SEG, and at an intermediate, but yet variable distance between the foramen and the lateral edge of the brain/SEG (Figure 6, B in majors; E, F in mediums and J, K in minors). At the bottom of the SEG, clusters c14 and c15 are located somewhat parallel to one another on a seemingly curve trajectory and symmetrically on both sides of the SEG (Figure 6, A). Cluster c15, is present as a row of cells closest to the inferior lateral edges of the SEG; while c14 is internal to c15. In minors, c14 and c15 are usually formed by a group of three cells each (Figure 6, M); however, sometimes a fourth cell could be observed in c14 (Figure 6, O). In medium workers both clusters (c14, c15) are more irregular in their bilateral spatial arrangement compared with minor workers, and cell numbers may vary from 1 to 3 in each cluster even within the same individual. For example, in a medium worker only one cell is seen in c15, and three are detected in c14 in left hemisphere (Figure 6, H,) while in the right hemisphere there are 3 cells in c15 with c14 showing only two cells (Figure 6, I). In majors cluster c14 is absent, but c15 is clearly visible very close to the lateral edge (Figure 6, B–D) and could be composed from 3 to 5 cells.

**Figure 7:**
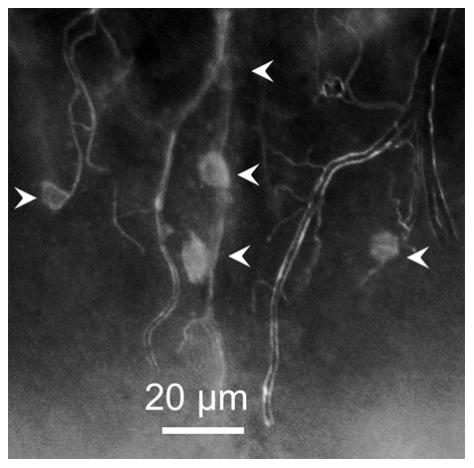
Cluster c16 is located near trachea in the posterior brain. This cluster is observed in all subcastes, generally located at the center of the SEG, with 5 cells distributed in a cross-like fashion. Sometimes, the location of each individual cell can vary, and they could be observed near, or perhaps in association with trachea, as shown here in a minor brain.

In all worker subcastes c16 is located at the center of the SEG. It is composed of a group of five cells, most commonly distributed in a cross-like fashion (Figure 6, A, E, G) sometimes showing some distortion in the cross-like distribution as seen in Figure 6 B, L and N. These cells are very similar in shape and size, but usually two of them (center, bottom two cells in c16, Figure 6, G, L) appear with a higher fluorescence intensity compared with the other three. It is not known yet if this is due to a higher expression of the sNPF receptor, or as a consequence of the variable distance of these cells to the surface of the SEG. Negative controls did not show immunoreactivity (Figure 6, P, Q). In some instances these c16 cells appeared to be attached or in close proximity to trachea (Figure 7).

### Expression of the sNPFR on the brain/SEG of worker ants from colonies without brood

**Figure 8:**
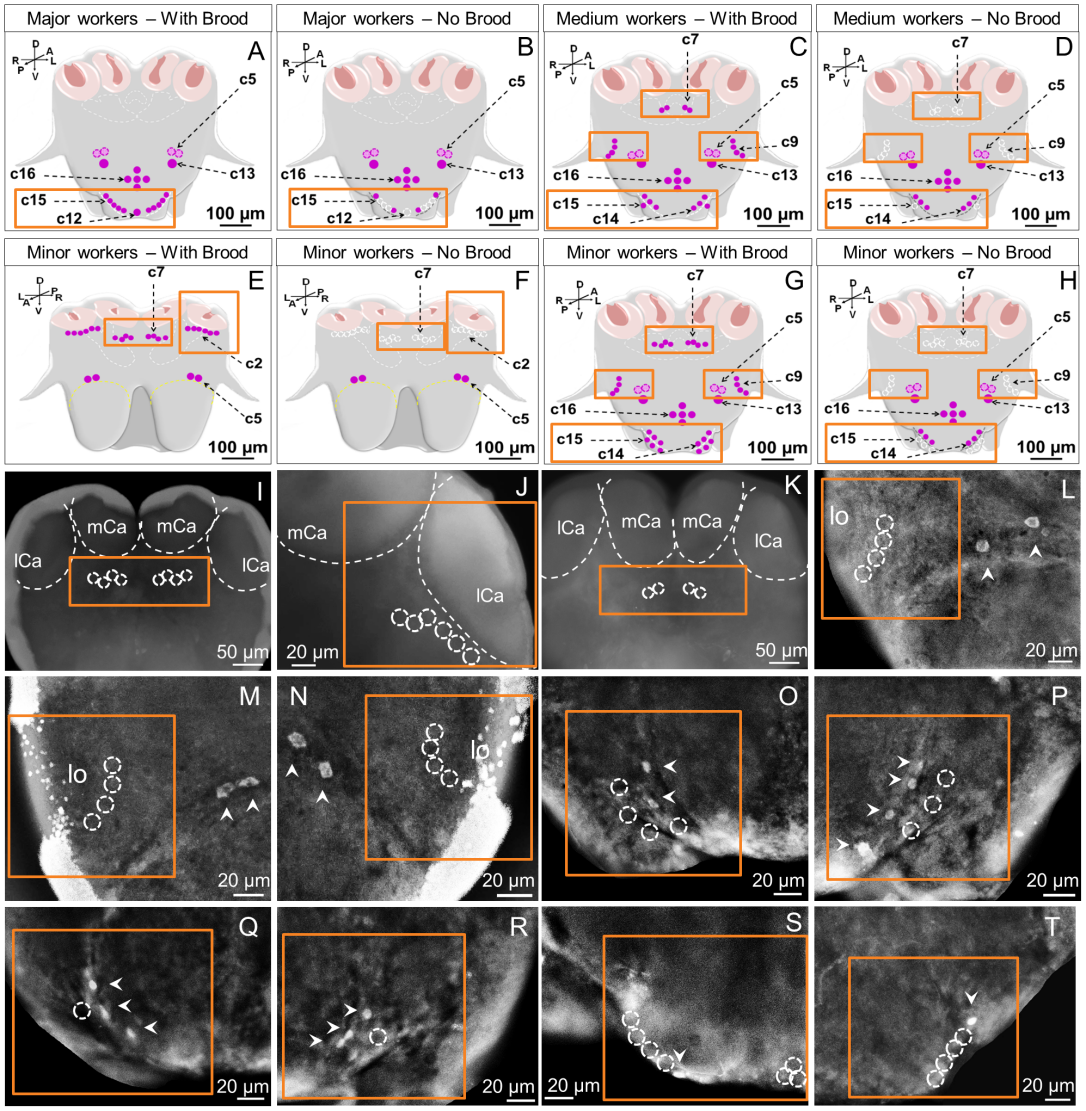
Brain immunolocalization of the sNPFR in worker subcastes from colonies without brood and comparisons with those with brood. (A–H) Schematic of the location of cell clusters expressing the sNPFR in all subcastes, comparing colonies with and without brood. Areas in orange boxes enclose specific brain areas for comparison. Dashed white circles correspond to the expected location of immunoreactive cells in colonies with brood. A–D, G and H show the receptor signal in the posterior brain; E and F show the anterior brain view. In colonies without brood cluster c7 became undetectable both in minor (H, I) and medium workers (D, K); compare with C, G. Additionally, cluster c2 became undetectable in minors from these colonies (F, J). In the posterior view, cluster c9 also became undetectable in minors (H, and insets in M, N) and mediums (D, L, inset). Cluster c14 remained present in minor (O, P) and medium workers (Q, R), but cluster c15 became undetectable in these workers, and only observed in majors, however, composed by lower number of immunoreactive cells (S, T) in comparison to majors in colonies with brood. Cluster c12 also is undetectable in majors (B, and in S, lower right corner) when brood is absent.

We investigated differences in the number of clusters and cells within clusters expressing the sNPFR that correlated with the presence/absence of brood in the colony (Figure 8, A–H). Some worker brain clusters remain unchanged with respect to colonies with brood. For example, in workers from colonies without brood clusters c5, c13 and c16 were detected in all worker subcastes in the same location and with the same characteristics mentioned above (data not shown and Figure 8, B, D, F, H), and c14 was also present in minor (Figure 8, H, O (left), P (right) and medium (Figure 8 D, Q (left), R (right) workers as before; c14 is never present in majors (Table 1).

For other clusters, in workers from colonies without brood the number of immunoreactive cells for the sNPFR was considerably reduced in comparison to workers of colonies with brood. This was most strikingly seen in the major-exclusive cluster c12 that became undetectable (Figure 8, B, S (right)) and in the worker caste ubiquitous c15 (Figure 8, B, D, H; O, P, minors; Q, R, mediums and S, T, majors) that remained only observable in majors (Figure 8, B, S, T) while being undetectable in minor (Figure 8, H, O, P) and mediums (Figure 8, D, Q, R) in colonies without brood. In majors, the number of cells in c15 was drastically reduced from 3–5 in colonies with brood to 1–2 cells in those without brood (Figure 8, compare A to B; and compare majors without brood S and T with c15 in Figure 6, B–D).

Two clusters, c7 and c9, were undetectable in medium and minor worker brains from colonies without brood. Cluster c7, normally composed of 2–3 cells in mediums (Figure 8, C) was not detectable (Figure 8, D, K) while in minors, normally composed of 4 cells (Figure 8, E, G), was also absent in colonies without brood (Figure 8, H, I). Cluster c9, was undetectable in both mediums (Figure 8, D, L; compare with Figure 5, C, H) and minors (Figure 8, H, M, N; compare with Figure 5, B, G) in colonies without brood. Cluster c2 normally only present in minors of colonies with brood (Figure 8, E, and Figure 3, J, L) was undetectable in those from colonies without brood (Figure 8, F, J).

### Differences among castes and subcastes in sNPFR cell clusters

In summary, sNPFR immunoreactive clusters detected in workers that are apparently in similar location in the queen brain are: c5 (also in all worker subcastes); c2, c7, c9 and, c12. Clusters c13-c16 are present exclusively in workers, of which c13, c15 and c16 are detected in all subcastes, the latter likely representing worker specific functions. About clusters and characteristics that discriminate subcastes: majors are the only subcaste that always lack c14 immunostaining but in which c12 is present in colonies with brood, while c12 is always absent in mediums or minors (Table 1). Minors can be distinguished because are the only subcaste that exhibit c2 in colonies with brood and this is a critical difference with mediums with which they share others clusters. In colonies with brood, mediums can be distinguished from majors in that mediums have c7, c9 and c14 immunoreactivity; and from minors, in that mediums lack c2 (Table 1). In colonies without brood majors can be distinguished from both mediums and minors in that majors retain c15 immunostaining, even when the number of cells is reduced, while mediums and minors cannot be discriminated by sNPFR staining in these colonies.

## Discussion

The spatial expression pattern of the sNPFR in the fire ant worker brain was analyzed in all subcastes, the first time this is reported for workers of a social insect. The sNPF and sNPFR have been characterized in *Drosophila* [31]–[34] where brain sNPF expression is broad; in contrast, in honey bee workers brain, only a few neurosecretory cells are labeled by sNPF *in situ* hybridization [25], pointing to striking differences between solitary and social insects. There is a paucity of information on this signaling system in other social insects other than honey bees. Therefore, our previous study on fire ant queen brains was now followed by the analyses on workers.

Our results are particularly important for fire ants because worker subcastes are loosely defined by age and size, with observed performed tasks varying with the individual's age creating temporal castes. Therefore, currently there are not known clear morphological or neurobiological markers for determination of worker subcaste as to precisely define the number of individuals most likely performing a particular task at a particular time in a colony. Behavioral plasticity adds to the impossibility of defining the division of labor precisely [35]. Although workers were not discriminated by age in this study, it appears that age variation may have been coincidentally minimized perhaps because ants were chosen of a particular size and performing a specific task; as a consequence, the cellular expression pattern of sNPFR remained quite constant within subcastes. We did not use ants of different sizes performing the same task (perhaps of different ages; i.e. an older small nurse now foraging); this is especially true for minors and majors. The overall decrease in sNPFR immunoreactivity in colonies without brood (Table 1) supports a relationship between sNPFR expression and colony higher nutritional status or requirements for protein when brood is present because of the known ability of larvae to digest protein [36].

In contrast to the paucity of information in fire ants, there is more knowledge about mechanisms regulating division of labor and behavioral plasticity in worker honey bees. In these bees, about 5,500 brain genes are differentially expressed between nurses and foragers [37], and it is also established that division of labor is related to feeding behavior, nutritional status and age of each individual [38]–[40]. Additionally, there is evidence that the sNPF/sNPFR signaling pathway regulates the foraging behavior in honey bees, and that the sNPFR transcript appears to be upregulated in the brain of foragers compared to nurses [25], and strikingly, the sNPF peptide level changes in association with nectar or pollen foraging, and between foragers arriving or departing from a feeder [26]. Pollen-collecting bees departing from feeders with pollen on their legs had higher sNPF peptide in their brains [26]. These findings are in agreement with our results with nurses in colonies with brood, in which we found a significant higher number of cells exhibiting sNPFR immunoreactivity in the brain/SEG. Similar to pollen-collecting bees, fire ant nurses handle protein, and feed proteins to larvae and queens preferentially; this is more evident when protein supplies are limited [41]. Although we have not yet investigated sNPF expression in fire ants, it seems that in bees and ants, protein sensing correlates with increased sNPF signaling effector proteins, sNPF or sNPFR, respectively.

Globally, the worker caste exhibited a total of 9 cell clusters expressing the sNPFR, with different number of clusters observed in the different subcastes (Figure 2). Clusters 13, 15 and c16 are present in all worker subcastes but not the queen, indicating that represent worker exclusive functions.

There is an inverse relationship between worker size and number of clusters expressing sNPFR, with majors having the lowest cluster number of five. Interestingly, the range in the total number of sNPFR cells did not overlap among subcastes in colonies with brood (Table 1) indicating that the sNPFR clusters indeed correlate with functional subcastes (size and task performed). The lesser total number of sNPFR immunoreactive neurons found in majors (19–26) correlates with their known simpler behavioral suite: only 15 behaviors were registered for majors compared to 20 for minors, in which we observed higher sNPFR immunoreactive cells [5]. Further, 72% of majors lifespan is spent as reserves and about 27% as foragers, with only a minimal (1% of life span) time caring for brood [6]. We observed that in majors cluster c15 decreased in the number of sNPFR stained cells in the absence of brood, perhaps also reflecting a “protein-starved” phenotype (Table 1), while immunoreactivity fully disappeared in mediums and minors.

Worker fire ants are completely sterile, offering a model in which queen reproductive functions could be differentiated from other functions in workers, contrary to the honey bee in which workers may retain reproductive ability [42]. With respect to this, some of the cell clusters found in the worker brain are also present in the fire ant queen brain, as described by Lu *et al* [28]; thus, it appears there are indeed common circuits and functions modulated by the sNPFR in workers and queens, such as exemplified by C5 (Figure 4, Table 1). Cluster c5 located above the antennal lobes (Figure 4), was present in all subcastes from colonies with and without brood. In the queen brain C5 was identified as possible local and/or projection interneurons, which would transmit the information generated on the olfactory receptor neurons (ORNs) to higher processing centers in the brain [28]. We also observed C5 in males (data not shown). This suggests that its function is independent of size, age and specific labor of the workers and it appears it has constitutive expression in all castes (Table 1).

As expected, others clusters were exclusive to one caste (see Table 1; C1-C12, queen exclusive clusters, and c13–16, worker caste exclusive), indicating there are pathways differentially regulated by this receptor in queens and workers. Further, the number of cells expressing the sNPFR in queens (∼164) is much higher compared to worker ants. This caste difference in sNPFR expression supports the notion that sNPF signaling is involved in regulation of additional complex functions in queens, such as reproduction, nutrient storage functions which queens display or others [28]. For example, cluster C1 present in the queen anterior protocerebrum which was postulated to correspond to insulin producing cells is not present in fire ant workers [28].

The most striking differences in the pattern of expression of the sNPFR among worker subcastes were found in the superior protocerebrum, below the mushroom bodies, and recent studies show the mushroom bodies seem to be involved in mechanisms related to the division of labor in ants [10], [11], [14], [16], bees [43]–[45] and a wasp [46]. In minor fire ants, cluster 2 (c2) located below the mushroom bodies is in similar location as in the queen brain (C2), and it was hypothesized that they could be neurosecretory, due to their similar location to the lateral neurosecretory cells (FMRFamide-like immunoreactive) in the honey bee brain [28]. Because C1 cells are not present in workers it would be interesting to know if C1 and C2 in queens and c2 in workers represent two separate functions of the sNPF signaling pathway, related and unrelated to insulin production in queens and workers, respectively. Similar to the central location of C1 in fire ant queens, in the *Drosophila* brain a cluster of centrally located cells, the medium neurosecretory cells, are insulin producing and express sNPFR, however, in the same cluster, a few sNPFR expressing cells do not appear to produce insulin peptides, setting a precedent for the separation of expression of sNPFR and insulin like peptides in the insect brain [47].

Cluster c2 immunoreactivity was only observed in minor workers extracted from colonies with brood (Figure 3 J, L); while immunoreactivity was absent in minors from colonies without brood (Figure 8, F, J). This suggests that c2 could control common functions in the brain of queens and the minor workers studied here (likely “nurses”), and this hypothetic function could be related to protein sensing associated with brood handling and care. This subcaste appears to be more sensitive to the presence/absence of brood than mediums and majors, with a 63–71% reduction in the number of sNPFR cells in minors from colonies without brood (Table 1).

In colonies with brood, with respect to cluster changes in the superior medial protocerebrum, cluster c7 was detected only in medium (Figure 3 E, F) and minor (Figure 3, H, K) workers; being undetectable in mediums (Figure 8, D, K) and minors (Figure 8, E, I) from colonies without brood (Table 1). This cluster was never observed in major workers. sNPFR immunoreactive cells in approximately the same location were named C7 in the fire ant queen brain [28], and both clusters are located in a similar area as the cluster of octopaminergic neurons G4d, in the honey bee brain [48]. Due to the location of c7 near the central complex, we hypothesized the sNPF/sNPFR signaling pathway could also be regulating the function of this neuropil in the ant brain. In *Drosophila*, cells expressing the sNPF and the sNPFR are in the central complex [49], [50], and a reduction of sNPF expression in the fan-shaped body of female flies increases their walking distance and their mean walking speed [51]. The presence of c7 in minors and mediums (see Table 1) is also consistent with perhaps higher protein transfer near the nest when brood is present [29]. If c7 is involved in locomotion, it would be interesting to test if the immunoreactivity of these c7 cells changes with age in smaller workers as an age related, temporal polyethism, associated with foraging and the leaving of the nest in older workers.

Similar to the cluster c7, cluster c9 appears to be immunoreactive only in minors and medium workers from colonies with brood. In the queen brain C9 could correspond to optical projection neurons, similar to the ones observed in the ant *Cataglyphis albicans* [28], [52]. We speculate that in fire ants these cells are involved with the regulation of visual input and brood care.

Most clearly seen from the posterior view of the brain, clusters c12 to c16 are located in the inferior protocerebrum and in the subesophageal ganglion (SEG) (Figure 6). We hypothesized they are involved in the regulation of functions that are processed by the SEG, such as regulation of movement of the mouthparts [53], [54], gustation [55] and feeding behavior [56]. In different insect species including social insects several neuropeptides had been detected in the SEG, such as FMRFamide-like peptides, FXPLRamide related peptides (e.g. PBAN), neuropeptide F (NPF) and short neuropeptide F (sNPF) [33], [57], [58]. The expression pattern of the sNPFR in the SEG varies slightly among worker subcastes as shown under results (Figure 6), however, dramatic differences were observed in the SEG when comparing workers from colonies with and without brood (Figure 8). In the latter workers the number of sNPFR immunoreactive cells is considerably reduced specifically in clusters c15 (Figure 8, O–T) and c12 (Figure 8, B, S), which apparently could be due to a diminished nutritional status, maybe representing a mechanism of protein sensing. Clusters C12 is reminiscent of the octopaminergic ventral unpaired medium (VUM) neurons described in honey bee forager (worker) brains [48], [59]; perhaps these cells integrate foraging behavior for protein in majors. Octopamine is broadly expressed in the SEG of honey bees and the fire ant sNPFR signal overlapping with known patterns of octopaminergic neurons in worker bees points perhaps to the integration of olfactory (VUM, c12) and nutritional signals (sNPF/sNPFR) and olfactory learning in ants [48].

Based on the fact that a) majors are not associated behaviorally with brood care but exhibit c12, c13, c15 and c16, and that b) some clusters were present in all castes (C5), it is reasonable to assume that the mentioned clusters are not particularly associated with the presence or absence of brood as it pertains to brood presence per se. Among these, clusters that appear to be protein sensing are c15 and C12. It appears that different subcastes may perceive the lack of protein differentially through these clusters (depending on their worker subtask priority, i.e. nurses being more sensitive through cluster c2). The queen exhibits sNPFR immunoreactivity in 12 clusters, the highest cluster number than any worker caste, giving her perhaps a higher sensitivity to either protein availability/requirements or presence of brood. Further, queens and larvae are always preferentially fed protein even when protein is limiting [60]–[62]. Therefore, it follows that the queen may always have sNPFR immunoreactivity in these clusters.

## Conclusions

Dynamic changes in sNPFR appear to occur in worker brains in association to the presence/absence of brood, specifically in the protocerebrum and the SEG. Our study supports our previous work with starved fire ant queens in that the sNPFR signaling may change in response to nutritional requirements, not only in queens but workers. To our knowledge this is the first if not one of the few studies with social insects that correlate body size, behavioral state and colony brood presence (proxy for colony nutritional status/requirements) with the actual protein expression of a brain neuropeptide receptor, sNPFR, and not a transcript. In the absence of brood, the overall decrease in the sNPFR signal observed in worker brains supports the idea that workers may be less motivated to search for food. It is apparent that in fire ant workers, similar to bees, changes in sNPFR expression depend at least partially on foraging needs.
